# Can WRKY transcription factors help plants to overcome environmental challenges?

**DOI:** 10.1590/1678-4685-GMB-2017-0232

**Published:** 2018

**Authors:** Taciane Finatto, Vívian Ebeling Viana, Leomar Guilherme Woyann, Carlos Busanello, Luciano Carlos da Maia, Antonio Costa de Oliveira

**Affiliations:** ^1^ Centro de Genômica e Fitomelhoramento, Departamento de Fitotecnia, Faculdade de Agronomia Eliseu Maciel, Universidade Federal de Pelotas, Pelotas, RS, Brazil; ^2^ Programa de Pós-Graduação em Biotecnologia, Centro de Desenvolvimento Tecnologico, Universidade Federal de Pelotas, Pelotas, RS, Brazil

**Keywords:** Transcriptional regulation, signaling, abscisic acid, kinases, stresses

## Abstract

WRKY transcription factors (TFs) are responsible for the regulation of genes responsive to many plant growth and developmental cues, as well as to biotic and abiotic stresses. The modulation of gene expression by WRKY proteins primarily occurs by DNA binding at specific *cis*-regulatory elements, the W-box elements, which are short sequences located in the promoter region of certain genes. In addition, their action can occur through interaction with other TFs and the cellular transcription machinery. The current genome sequences available reveal a relatively large number of WRKY genes, reaching hundreds of copies. Recently, functional genomics studies in model plants have enabled the identification of function and mechanism of action of several WRKY TFs in plants. This review addresses the more recent studies in plants regarding the function of WRKY TFs in both model and crop plants for coping with environmental challenges, including a wide variety of abiotic and biotic stresses.

## Introduction

Plants are continuously exposed to environmental stresses, such as variations in temperature, nutrient content and availability in the soil, rain availability, and pest/pathogen attacks. Their exposure to this range of stresses unleashes responses at various levels. At the molecular level it activates signaling cascades responsible for inducing or repressing target gene expression. Transcription factors (TFs) are proteins that regulate gene transcription, and any change in their activity dynamically alters the transcriptome, causing metabolic and phenotypic changes in response to a given environmental stimulus (Mitsuda and Ohme-Takagi, 2009).

In eukaryotes, the transcriptional regulation is mediated by recruitment of TFs that recognize and bind to *cis*-regulatory elements (CREs), which are short sequences present in the promoter regions of genes. TFs interact with CREs, other TFs, and with the basal transcription machinery in order to regulate the expression of target genes (Priest *et al.*, 2009).

Stimuli caused by environmental changes are primarily perceived by a receptor and later transmitted to the nucleus by a complex network of protein interactions. The signals can be transmitted to the nucleus by several systems, including GTP-binding (G proteins that alter their activity by binding to GTP), protein kinase cascades that are phosphorylated in sequence and activate a series of proteins, and ion channels that change ionic features of cells (Mulligan *et al.*, 1997). As observed in the majority of adaptive responses, gene expression is strictly controlled and presents a fast and reversible kinetic response, allowing a cell to change its transcriptional status within minutes in the presence of the stressor, and to return to its basal state when that is no longer present (de Nadal *et al.*, 2011).

Eukaryotic cells have developed sophisticated mechanisms of sensitivity and signal transduction systems that can produce precise results and dynamic responses. Receptor molecules are stimulated by external stimuli and initiate a cascade of downstream signaling network that, through a cross-talk with molecular cell process, respond to the various environmental and developmental stimuli (Osakabe *et al.*, 2013). Changes in gene expression are an important component of stress responses, together with metabolic changes, cell cycle progression, cytoskeleton protein homeostasis, vesicle protein trafficking, enzymatic activity (de Nadal *et al.*, 2011), production of reactive oxygen species (Baxter *et al.*, 2014), and protein phosphorylation (Shiloh and Ziv, 2013).

WRKY factors play an essential role in the regulation of many stress responses of plants, and many studies related to this class of TFs have described activity in response to biotic stresses. However, some recent reports have shown their link also to abiotic stresses. Many WRKY genes have roles in multiple pathways induced by stresses, suggesting nowadays that the signal network involving WRKY includes biotic and abiotic stresses (Eulgem, 2006). However, little is known about the signaling pathways mediated by WRKY TFs induced by abiotic stresses (Rushton *et al.*, 2010).

Most WRKY proteins are involved in responses to bacterial and fungal pathogens, hormones related to pathogens, and salicylic acid (Rushton *et al.*, 1996; Eulgem *et al.*, 1999). They usually mediate signaling by the elicitor molecule encoded by the pathogen and quickly induce apoptosis of cells so as to avoid posterior invasion (Yamasaki *et al.*, 2005).

An increasing number of recent studies have indicated that WRKY proteins are also involved in a variety of other plant specific reactions such as senescence (Miao *et al.*, 2008), mechanical damage (Hara *et al.*, 2000), drought (Zhang *et al.*, 2008; Liu *et al.*, 2011), heat shock (Li S *et al.*, 2010) high salinity (Wang F *et al.*, 2012; Niu *et al.*, 2012), UV radiation (Izaguirre *et al.*, 2003), sugar signaling (Sun *et al.*, 2003), gibberellins (Zhang *et al.*, 2004), abscisic acid (ABA) (Xie *et al.*, 2005; Chen *et al.*, 2010), and many other processes (reviewed by Agarwal *et al.*, 2011; Phukan *et al.*, 2016). Several studies concerning genetic transformation, including generation of transgenic lines, aiming to identify WRKY TFs functions in response to various biotic and abiotic stresses are shown on supplementary material (Tables S1, S2, S3 and S4).

Given the importance of this transcription factor family and its relation to biotic and abiotic stresses, the objective of this review was to survey the current knowledge on the regulation, activity, and targets of WRKY family TFs, as well as to give an overview of the main studies about losses and gains of function of WRKY genes in different plant species.

## The structure of WRKY transcription factors

WRKY proteins share a DNA binding domain of about 60 amino acids that contains an invariable sequence WRKYGQK (from which the domain was named - WRKY) and a zinc-finger domain-like (CX_4_–_5_CX_22–23_HXH or CX_7_CX_23_HXC) (Rushton *et al.*, 1996). Many WRKY proteins have two WRKY domains, which are classified in Group I. Those that have a single WRKY domain and containing the zinc-finger motif Cys_2_-His_2_ are classified in Group II, which is divided into five subgroups (IIa-e) based on additional structural motifs conserved outside the WRKY domain. The proteins from Group III show a WRKY domain containing different zinc-finger motifs Cys_2_-His/Cys Cys_2_-His_2_ (Eulgem *et al.*, 2000).

A new WRKY structure has recently been reported, and different novel substitutes of WRKY domains in N- and C-terminal region of WRKY TFs were found (Mohanta *et al.*, 2016). The conserved WRKY amino acids were replaced by different types of amino acids in many different plant species compared to *Arabidopsis* e.g. W-K-K-Y in *Brassica rapa*, *Citrus clementine*, *Citrus sinensis*, *Eucalyptus grandis*, *Linum usitatissimum*, *Phaseolus vulgaris*, *Physcomitrella patens*, *Picea abies*, *Populus trichocarpa*, *Prunus persica* and *Sorghum bicolor*; and W-R-I-Y in *Arabidopsis lyrata*, W-H-Q-Y in *Glycine max*, A-R-K-M, W-W-K-N and W-R-M-Y in *Phaseolus vulgaris*, W-R-K-R, W-I-K-Y, W-S-K-Y and W-Q-K-Y in *Solanum lycopersicum*, W-H-K-C and W-R-K-C in *Solanum tuberosum*, F-R-K-Y in *Populus trichocarpa* (Mohanta *et al.*, 2016).

Subsequently, based on phylogenetic analyses, WRKY TFs were divided into I, IIa + IIb, IIc, IId + IIe, and III. Group II was found to be monophyletic (Zhang and Wang, 2005). Besides the two highly conserved domains, WRKY and zinc-finger, Cys2-His2 or Cys_2_-His/Cys WRKY proteins also contain the following structures: basic signals of putative nuclear localization, leucine zipper, and TIR-NBS-LRR kinase domains that are serine-threonine, glutamine or proline abundant regions. These domain variations in their structure make WRKY proteins play their appropriate roles in the regulation of gene expression (Chen *et al.*, 2012). WRKY proteins are known to mediate signaling by binding to the target gene promoter regions that contain W-box (T) TTGACY sequences, where the letter Y can mean C or T (Pater *et al.*, 1996; Rushton *et al.*, 1996; Eulgem *et al.*, 1999). Genes containing the ERAC W-box in their promoter regions are targets of WRKY including the genes from WRKY family (Eulgem *et al.*, 1999).

WRKY TFs were originally considered exclusive to plants. However, recent reports have detected their presence also in protists (*Giardia lamblia*) and in Metazoa (*Dictyostelium discoideum*), suggesting an origin of WRKYs predating the origin of plants (Ulker and Somssich, 2004).

A survey of sequenced plant genomes has revealed that the WRKY TF family is composed by a large number of genes, i.e., 74 genes in *Arabidopsis thaliana*, 81 genes in *Brachypodium distachyon*, 51 in *Citrus sinensis,* 48 in *Citrus clementina*, 38 genes in *Daucus carota*, 179 genes in *Glycine max*, 58 genes in *Jatropha curcas*,117 genes in *Manihot esculenta*, 123 genes in *Malus domestica*, 54 in *Morus notabilis*, 116 genes in *Oryza sativa* ssp. *Indica*, 137 genes in *Oryza sativa* ssp. *japonica*, 88 genes in *Phaseolus vulgaris*, 119 genes in *Populus trichocarpa*, 79 genes in *Solanum lycopersicum*, 82 genes in *Solanum tuberosum,* 98 genes in *Vitis vinifera*, and 180 genes in *Zea mays*, (Zhang *et al.*, 2010; Xiong *et al.*, 2013; Ayadi *et al.*, 2016; Baranwal *et al.*, 2016; Li *et al.*, 2016; Mohanta *et al.*, 2016; Liu *et al.*, 2017).

## WRKY TF and kinase interaction

Mitogen activated proteins kinases (MAPKs) modulate many plant signaling responses, including response to stresses, making a connection between stimuli perception and molecular cell responses (de Zelicourt *et al.*, 2016). Also, pathway signaling activation mediated by MAPKs is a vital process for plant responses (Sheikh *et al.*, 2016).

**Figure 1:**
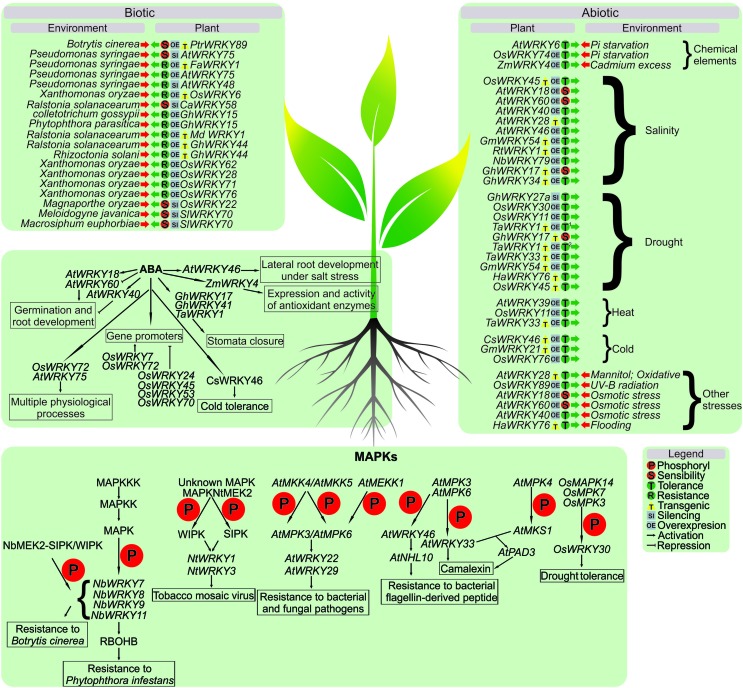
Overview of the most recent reports concerning WRKY roles in plant defense, including the relationships with MAPKs, ABA signaling, and responses to biotic and abiotic stresses.

Several studies have suggested that certain WRKY proteins can be targeted by MAPKs, which alter their activity (Figure 1). For example, WIPK (wound-inducible protein kinase) and SIPK (salicylic acid-inducible protein kinase) are two widely studied kinases concerning stress response. They are phosphorylated by MAPKNtMEK2 (in tobacco) in response to infection by a pathogen and by an unknown MAPK upstream. Both WIPK and SIPK seem to be upstream of *NtWRKY1* and *NtWRKY3* in the signaling cascade of Nicotiana’s defense response (Kim and Zhang, 2004). *AtWRKY22* and *AtWRKY29* were also identified as located downstream in the MAPK signaling cascade (Asai *et al.*, 2002).

The mitogen activated protein kinase genes *MPK3* and *MPK6* are responsive to pathogens and play an essential role in inducing camalexin, the main phytoalexin in *Arabidopsis thaliana* (Han *et al.*, 2010). *Arabidopsis* mutants in the *wrky33* gene with gain of MPK3 and MPK6 function showed impairment in the production of camalexin induced by pathogens. AtWRKY33 is a transcription factor induced by pathogens whose expression is regulated by the MPK3/MPK6 cascade. Furthermore, immunoprecipitation experiments showed that AtWRKY33 binds to its own promoter *in vivo*, suggesting a possible regulation by means of a feedback loop (Mao *et al.*, 2011). The AtWRKY33 protein is the substrate of MPK3/MPK6, and mutations in phosphorylation sites of MPK3/MPK6 in AtWRKY33 compromise their ability to complement the induction of camalexin in *Atwrky33* mutants. Additionally, in assays of phosphoprotein mobility, it was observed that AtWRKY33 is phosphorylated *in vivo* by MPK3/MPK6 in response to infection by *Botrytis cinerea*. AtWRKY33 acts downstream of MPK3/MPK6 in reprogramming camalexin biosynthesis genes, boosting metabolic flow towards the production of *Arabidopsis* camalexin in response to pathogens (Mao *et al.*, 2011). Likewise, another report demonstrates a relationship of AtWRKY33 with a MAPK (Qiu *et al.*, 2008). In the *Atwrky33* mutant, a reduction of *PAD3* (*Phytoalexin deficient3*) mRNA was detected, which was also observed in a *mpk4-wrky33* double mutant. This suggested that AtWRKY33 acts with MPK4 in nuclear complexes that depend on MKS1, an MPK4 substrate. When infected with *Pseudomonas syringae*, MPK4 is activated and phosphorylates MKS1, which binds to AtWRKY33. That complex binds to a PAD3 promoter, which itself encodes an enzyme for camalexin synthesis (Qiu *et al.*, 2008).

A recent report demonstrates the relationship of MPK3 and MPK6 in targeting WRKY phosphorylation in 48 *Arabidopsis* WRKYs *in vitro*. Most of the analyzed WRKYs were targets of both MPKs. A phosphorylation of AtWRKY46 was analyzed *in vivo,* and elicitation with bacterial flagellin-derived flg22 peptide (a pathogen associated molecular pattern - PAMP) led to *in vivo* AtWRKY46 responses. When overexpressed, it raised basal plant defense, as reflected by the increase in promoter activity of NHL10 (a PAMP-responsive gene), in a MAPK-dependent pathway. A mechanism of plant defense controlled by AtWRKY46 in a MAPK-mediated pathway was suggested (Sheikh *et al.*, 2016).

In rice it was demonstrated that overexpression of *OsWRKY30* transgenic lines improved drought tolerance. OsWRKY30 could interact with and is phosphorylated by OsMPK3, as well as by many other OsMAPKs. Moreover, phosphorylation of OsWRKY30 by MAPKs is crucial for its biological function (Shen *et al.*, 2012). *OsWRKY30*, in addition to the two WRKY domains in the N-terminus, presents multiple serine-proline (SP) sites that can be putatively phosphorylated by MAP kinases (Cohen, 1997). OsWRKY30 was found phosphorylated *in vitro* by OsMPK3, OsMPK7, and OsMPK14, suggesting that OsWRKY30 is a potential substrate of OsMPKs, which are proline-directed kinases (Shen *et al.*, 2012).

Studies in *Nicotiana benthamiana* indicated that phospho-mimicking mutations of WRKYs result in strong induction of cell death, revelaed by examination of *WRKY 7-15* made by agroinfiltration in leaves. Four days after the infiltration, trypan blue staining showed that *WRKY 7, 8, 9, 11, 12*, and *14* were expressed in leaves developing cell death, suggesting that these WRKYs are involved in inducing cell death as MAPK substrate (Adachi *et al.*, 2015). In addition, VIGS-silencing construction with WRKY7, 8, 9, and 11 in leaves delayed cell death in VIGS of SIPK, and WIPK; a suppression of MEK2^DD^ was detected. Difference between TRV-control and WRKY7, 8, 9, and 11-silenced leaves was found through an index of cell death suggesting that this WRKYs participates in HD-like cell death downstream of a MEK2-SIPK/WIPK cascade (Adachi *et al.*, 2016).

## WRKY and abscisic acid signaling

Abscisic acid (ABA) plays a variety of roles in plant development, bud and seed dormancy, germination, cell division and movement, leaf senescence and abscission, and cellular response to environmental signals (Rohde *et al.*, 2000; Leung and Giraudat, 1998; Zhu, 2002; Xie *et al.*, 2005). Many reports have shown the involvement of WRKYs and ABA (Figure 1). During drought stress, the accumulation of ABA leads to stomata closure, which helps to maintain the cell water status under water deficit conditions by reducing water loss as a result of transpiration (Schroeder *et al.*, 2001). The possibility that WRKY TFs are involved in stomata closing as a response to both biotic and abiotic stress is an area that requires more research, although evidence is slowly appearing (Rushton *et al.*, 2012).

In *Arabidopsis*, WRKY proteins act as transcription activators or repressors. Through single, double, and triple mutants, as well as lines overexpressing *WRKY* genes analyses, it was shown that AtWRKY18 and AtWRKY60 have positive effects on the ABA sensitivity of plants regarding inhibition of seed germination and root growth and increase in the sensitivity to salt and osmotic stress. *AtWRKY40* antagonizes with *AtWRKY18* and *AtWRKY60* concerning the effect caused on the sensitivity of plants to ABA and abiotic stresses, and both genes are rapidly induced by ABA, while induction of *AtWRKY60* is delayed by ABA (Chen *et al.*, 2010). ABA-regulated genes such *REGULATORY COMPONENT OF ABA RECEPTOR* (*RCAR*), *ABA INSENSITIVE 1* (*ABI1*) and ABI2, together with *ABSCISIC ACID RESPONSIVE ELEMENTS-BINDING FACTOR* (*ABF*) were found differentially expressed in the *Atwrky40* mutant indicating that they are directly regulated by *AtWRKY40* (Shang *et al.*, 2010). On the other hand, *AtWRKY18*, *AtWRKY60* and *AtWRKY40* were reported as negative regulators of *ABI4* and *ABI5* genes by binding to W-box sequences in their promoters suggesting a negative role in ABA signaling (Liu *et al.*, 2012).

In addition, expression of *AtWRKY60* induced by ABA is practically null in the mutants *Atwrky18* and *Atwrky40*, indicating that AtWRKY18 and AtWRKY40 recognize a group of W-box sequences in the promoter of *AtWRKY60* and activate the expression of *AtWRKY60* in protoplasts. Thus, *AtWRKY60* could be a direct target of AtWRKY18 and AtWRKY40 in ABA signaling (Chen *et al.*, 2010).

Stomata closure was also tested in tobacco by overexpressing lines, showing that ABA participates in *GhWRKY41*-induced stomata closure. Stomata movement was analyzed with or without ABA treatment, and the authors found that ABA treatment reduced stomata apertures in the wild type and overexpression lines (Chu *et al.*, 2015).

In rice aleurone cells, the genes *OsWRKY24* and *OsWRKY45* acted as repressors of a gene promoter induced by ABA, while *OsWRKY72* and *OsWRKY7* activated the same gene promoters (Xie *et al.*, 2005). Reports on *OsWRKY53* and *OsWRKY70*, paralogs of *OsWRKY24*, showed the same effect, acting as repressors of a gene promoter induced by ABA (Zang *et al.*, 2015). In *Arabidopsis*, *OsWRKY72* interfered in the abscisic acid signal and auxin transport pathway, so that *Arabidopsis* lines overexpressing *OsWRKY72* showed retarded seed germination under normal conditions and higher sensitivity to mannitol, NaCl, ABA stresses, and sugar starvation when compared to vector plants (Yu *et al.*, 2010).

## WRKY and response to abiotic stresses

The relationship between WRKYs in response to abiotic stress has been investigated and is shown in Figure 1 and Table S1. Most reports describe the overexpression of different *WRKY* genes that resulted in abiotic stress-tolerant phenotypes in different plant species. The majority of stresses studied were salt excess, heat, and drought.

### Chemical elements

In *Arabidopsis*, the regulation of the *PHOSPHATE1* (*PHO1*) gene, which encodes for a phosphate carrier protein from root to shoot (Hamburger *et al.*, 2002), is also important for the adaptation of plants to low phosphate environments (Chen *et al.*, 2009). Under normal conditions, *AtWRKY6* suppresses the expression of *AtPHO1* to maintain homeostasis of phosphate in the plant through binding to two W-box CREs located in the *AtPHO1* gene promoter region (Chen *et al.*, 2009). Regarding phosphate starvation in rice, *OsWRKY74* was reported to be involved in tolerance mechanisms. When overexpressed, root and shoot biomass and phosphorus concentration were higher compared to wild type, in an opposite way from what was found in *OsWRKY74* loss of function (Dai *et al.*, 2016).

Cadmium stress studies in maize revealed the involvement of *ZmWRKY4* in response to stress. *ZmWRKY4*-silencing by RNAi in mesophyll protoplasts reveal its involvement as a requirement in ABA-induced genes expression and in antioxidative enzyme activity. In overexpression lines, ABA-induced genes were up-regulated and antioxidative enzyme activity was promoted (Hong *et al.*, 2017).

Transcriptomic analyses have also revealed the involvement of WRKYs in nutrient stresses. In rice leaves under manganese stress, six up-regulated WRKYs were identified suggesting their involvement in the stress (Li *et al.*, 2017). Involvement of WRKYs was also detected through microarray analysis in rice leaves under iron stress (Finatto *et al.*, 2015).

### Salinity

To study the response to salinity, desiccation, and oxidative stress, *Arabidopsis* transgenic lines expressing a multigene cassette with *GUS*, *AtWRKY28,* and *AtBHLH17* (*AtAIB*) were developed. *AtbHLH17* (*AtAIB*) and *AtWRKY28* are TFs known to be up-regulated under drought and oxidative stress, respectively. The transgenic lines exhibited enhanced tolerance to NaCl, mannitol, and oxidative stress. Under mannitol stress condition, significantly higher root growth was observed in transgenic plants. Growth under stress and recovery growth was substantially higher in transgenic plants exposed to gradual longer desiccation stress conditions. The TFs AtNAC102 and AtICE1, which contain either bHLH or WRKY binding *cis*-elements, showed enhanced expression in transgenic plants under stress. However, genes lacking either one of the two motifs did not differ in their expression levels in stress conditions when compared to wild type plants (Babitha *et al.*, 2012). Moreover, in *Arabidopsis*, overexpression of *AtWRKY46* enhanced lateral root development in salt stress via regulation of ABA signaling. In ABA-related mutants, *AtWRKY46* expression was down-regulated but up-regulated by an ABA-independent signal induction under salt stress (Ding *et al.*, 2015).

Another report regarding salinity showed that transgenic *Arabidopsis* plants overexpressing *GhWRKY34* (*Gossypium hirsutum*) enhanced the tolerance to salt stress by improving the plants’ ability to selectively uptake Na^+^ and K^+^ and maintain low Na^+^/K^+^ in leaves and roots of transgenic plants (Zhou *et al.*, 2015). Transgenic *Arabidopsis* plants overexpressing *GmWRKY54*, a soybean WRKY, also displayed salt tolerance (Zhou *et al.*, 2008). Overexpressing lines had 70% of survival under 180 mM NaCl treatment while wild type plants showed a survival of 25%.

Furthermore, transgenic *Arabidopsis* overexpressing *RtWRKY1* (Rt: *Reaumuria trigyna*) also demonstrated salinity tolerance. Transgenic lines were able to develop roots, increase fresh weigh under salt stress, as well as showing an increase in antioxidative enzymes activity, and lower Na^+^ content and Na^+^/K^+^ ratio compared to wild type plants. Also, an enhanced expression of *RtWRKY1* was detected in plants treated with ABA (Du *et al.*, 2017).

Salinity stress was also tested in recombinant *Escherichia coli* cells overexpressing a *J. curca* WRKY. Recombinant cells showed tolerance with significant cell growth under NaCl, mannitol, and KCl supplemented media (Agarwal *et al.*, 2014). It was suggested that these JcWRKY can regulate stress responsive genes and be used to enhance tolerance to abiotic stress.

A study in *N. benthemiana* overexpressing *NbWRKY79* showed promoted tolerant to salinity. A reduced accumulation of reactive oxygen species and increase in activity of antioxidant enzymes during salt treatment was also found. Furthermore, an enhanced ABA-inducible gene expression was detected (Nam *et al.*, 2017).

### Drought

In *Vitis vinifera* it has been shown by stress induction that *VvWRKY7*, *8* and *28* are up-regulated in response to drought stress by ABA accumulation, which triggers stomata closure, reducing water loss (Wang *et al.*, 2014). The same tolerance is shown in virus-induced gene silencing (VIGS) of *GhWRKY27a*, which enhances tolerance to drought stress in cotton (*Gossypium hirsutum*). In addition, *GhWRKY27a* expression was increased by ABA treatment (Yan *et al.*, 2015).

In tobacco, the ectopic overexpression of *TaWRKY1*, a wheat WRKY, conferred tolerance to drought, demonstrating that the transgenic tobacco plants exhibited development under stress and, when treated with ABA, the stomata closure rate was increased (Ding *et al.*, 2015). However, overexpression of *GhWRKY17* in transgenic *N. benthamiana* enhanced plant sensitivity to drought and salt stress. No effect of exogenous ABA treatment was detected, since the expression of ABA-inducible genes was also decreased in transgenic lines (Yan *et al.*, 2014).

Studies in transgenic *Arabidopsis* expressing *TaWRKY1* and *TaWRKY33*, a wheat WRKY, revealed tolerance to drought and/or heat stresses (He *et al.*, 2016). Under drought stress, both overexpressing lines showed higher germination rate compared to wild type. However, *TaWRKY33* seeds had higher germination rate than *TaWRKY1* and wild type plants. Regarding heat stress, *TaWRKY33* seeds showed a high survival rate when exposed to 45 °C for 5 hours. Differences between *TaWRKY1* and wild type plants were not reported (He *et al.*, 2016). A study in transgenic *Arabidopsis* overexpressing *GmWRKY54*, a soybean WRKY, also detected tolerance to drought stress (Zhou *et al.*, 2008). Plants were withheld from water from 18 days to 3 weeks. When water treatment was recovered, transgenic lines showed up to 85% of survival while wild type plants showed only 30%.

In rice (cv. Sasanishiki), fusion of the gene promoter induced by heat shock *HSP101* with the *OsWRKY11* gene resulted in overexpression of *OsWRKY11* and increased tolerance to drought and heat (Wu *et al.*, 2009).

### Heat and cold

Heat stress was also studied in the *Arabidopsis* for *AtWRKY39* gene, which positively regulates the signaling pathways activated by salicylic (SA) and jasmonic (JA) acids and mediates responses to heat stress (Li S *et al.*, 2010). These authors performed a study with mutants featuring the silenced *AtWRKY39* gene, and they found an increase in sensitivity to heat stress, a reduction in germination, and a reduction in survival when compared with non-mutated wild type. On the other hand, mutants overexpressing *AtWRKY39* had increased heat tolerance compared to the wild type. In this study, the authors also verified the influence of *AtWRKY39*-induced silencing and overexpression on the expression of other genes. In *Atwrky39* plants, *AtPR1* (regulated by SA) and *AtMBF1c* (related to SA) genes showed lower regulation. On the other hand, the overexpression of *AtWRKY39* increased the expression of these genes.

Regarding cold stress, a study in *Arabidopsis* transgenic lines overexpressing *CsWRKY46*, a cucumber WRKY, showed higher survival rates of freezing seedlings at 4 °C. Hence, tolerance is suggested to be an ABA-dependent process, since an effect of exogenous ABA in transgenic lines was observed (Zhang *et al.*, 2016). Another study with transgenic *Arabidopsis* overexpressing *GmWRKY21*, a soybean WRKY, also demonstrated enhanced tolerance to cold stress (Zhou *et al.*, 2008). Overexpressing lines were able to survive after 80 minutes under -20 °C, showing a better growth and survival compared to wild type plants.

In rice, overexpression of *OsWRKY76* improved tolerance to cold stress at 4 °C (Yokotani *et al.*, 2013). Overexpressing plants showed a significant lower content of ion leakage compared to wild type plants until 72 hours under treatment, demonstrating membrane stability in the overexpressing lines. However, overexpressing lines showed an increased susceptibility to blast fungus (*Magnaporthe oryzae*), with severe symptoms when infected (Yokotani *et al.*, 2013).

Through transcriptomic analyzes, the involvement of WRKYs in low temperature stress have also been revealed. In paper mulberry cultivated under 4 °C, WRKYs were the second TFs family with expressive changes, comprising 71 genes differentially expressed, for the most part down-regulated (Peng *et al.*, 2015). Hence, the authors suggested that, together with AP2/ERF, bHLH, MYB, and NACs, WRKYs display a central and important role against cold in paper mulberry.

### Other stresses

Studies in transgenic *Arabidopsis* expressing *HaWRKY76*, a sunflower WRKY, were carried out demonstrating tolerance to flooding (complete submergence) and drought stresses (Raineri *et al.*, 2015). Transgenic plants showed higher biomass, seed production and sucrose content compared to the wild type plants in standard conditions. In addition, when tested under flooding condition, these plants demonstrated tolerance through carbohydrate preservation. In drought stress, the tolerance occurred by stomata closure and via ABA-dependent mechanism. Under both stresses, an increase in seed yield was reported (Raineri *et al.*, 2015).

In rice, *OsWRKY89* was reported to confer tolerance to UV-B radiation. When overexpressed, the SA levels were increased, which enhanced the tolerance to UV-B radiation, but also enhanced resistances to pathogen and pest attacks as to blast fungus and white-backed planthopper (Wang *et al.*, 2007).

## WRKY and response to biotic stress

WRKY genes are involved in gene signaling for resistance responses to biotic stresses, such as viruses, bacteria, fungi, insects, and nematodes (Figure 1 and Table S2). The defense mechanisms are controlled by signaling molecules, such as salicylic acid (SA), jasmonic acid (JA), and ethylene, or by combinations of these signaling compounds (Schroeder *et al.*, 2001). SA accumulates locally in infected leaves, as well as in non-infected systemic leaves after infection with biotrophic pathogens, and mediates the induced expression of defense genes, resulting in an enhanced state of defense known as systemic acquired resistance (SAR) (Ryals *et al.*, 1996; Adachi *et al.*, 2015).

A study with signal transduction in SA signaling in *Arabidopsis*, reported that AtWRKY28 and AtWRKY46 are transcriptional activators of *AtICS1* (coding for isochorismate synthase, an enzyme that acts in the conversion of chorismate to isochorismate in SA biosynthesis) and *AtPBS3* (*AVRPPHB SUSCEPTIBLE 3*) that plays an important role in SA metabolism (Schroeder *et al.*, 2001). Expression studies with *ICS1* promoter::*β*-glucuronidase (*GUS*) genes in protoplasts co-transfected with *35S*::*WRKY28* showed that overexpression of *AtWRKY28* resulted in a strong increase in GUS expression. Moreover, RT-qPCR analyses indicated that the endogenous *AtICS1* and *AtPBS3* genes were highly expressed in protoplasts overexpressing *AtWRKY28* or *AtWRKY46*, respectively. Electrophoretic mobility shift assays identified potential WRKY28 binding sites in the *AtICS1* promoter positioned at 445 and 460 base pairs upstream of the transcription start site. Mutation of these sites in protoplast transactivation assays showed that these binding sites are functionally important for activation of the *AtICS1* promoter. Chromatin immunoprecipitation assays with hemagglutinin-epitope-tagged WRKY28 showed that the region of the *AtICS1* promoter containing the binding sites at 445 and 460 bp was highly enriched in the immunoprecipitated DNA (Schroeder *et al.*, 2001).

In the same way, in transgenic lines of *Arabidopsis* overexpressing *VqWRKY52* (Vq- *Vitis quinquangularis*), resistance to pathogen was found (Wang *et al.*, 2017). *VqWRKY52* expression was induced by SA but not by JA treatment*.* Ectopic expression in *Arabidopsis* led to resistance to powdery mildew and *Pseudomonas syringae* pv*. tomato* DC3000, but increased susceptibility to *Botrytis cinerea* (Wang *et al.*, 2017). It was reported that *AtWRKY57* acts in an opposite way in *Arabidopsis*, acting as a negative regulator against an infection depending of JA signaling pathway (Jiang and Yu, 2016). Loss of function of *AtWRKY57* enhanced resistance against *Botrytis cinerea* infection. As shown by chromatin immunoprecipitation experiments, AtWRKY57 regulates positively the transcription of *JASMONATE ZIM-DOMAIN1* (*AtJAZ1*) and *AtJAZ5*, which encodes two important repressors of the JA signaling pathway by binding directly to their promoters (Jiang and Yu, 2016).

The modulation of resistance against *Rhizoctonia solani* in rice, which causes sheath blight disease, was discovered to be controlled by *OsWRKY80*. *OsWRKY80* expression was induced by *R. solani* infection, JA, and ethylene. *OsWRKY80* overexpression and silencing by RNAi, conferred resistance and sensibility, respectively, in response to sheath blight disease through the positive regulation of *OsWRKY4* by binding to their W-box or W-box-like sequences (Peng *et al.*, 2016).

NaWRKY3 is required for the elicitation of *NaWRKY6* by fatty acid conjugated with amino acids present in the oral secretions of larvae of *Manduca sexta*. On the other hand, silencing of one or both genes made plants highly vulnerable to herbivores, possibly indicating that both WRKY genes help the plants to differentiate mechanical damage from herbivore attacks (Skibbe *et al.*, 2008).

The *AtWRKY23* response to nematode infection in *Arabidopsis* has been reported. Silencing of *AtWRKY23* resulted in low infection of cystic nematode *Heterodera schachtii* (Grunewald *et al.*, 2008). In *Arabidopsis*, the heterologous overexpression of *OsWRKY23* increased the defense response to *Pyricularia oryzae Cav* and SA, besides increasing the expression of pathogenesis-related genes (PR) and providing increased resistance to the pathogenic bacteria *Pseudomonas syringae* (Shaojuan *et al.*, 2009). Studies on overexpression of *WRKY* genes furthermore indicated that these lines, besides displaying a particular pathogen resistance, also showed tolerance to abiotic stresses (Table S3).

In *Arabidopsis*, the constitutive overexpression of the *OsWRKY45* transgene confers a number of properties to transgenic plants. These properties include significantly increased expression of *PR* genes, enhanced resistance to the bacterial pathogen *Pseudomonas syringae tomato* DC3000, enhanced tolerance to salt and drought stresses, decreased sensitivity toward ABA signaling during seed germination and post-germination processes, and modulation of ABA/stress-regulated genes during drought induction. In addition, higher levels of *OsWRKY45* expression in transgenic plants correlate positively with the strength of the abiotic and biotic responses mentioned above. More specifically, the decreased ABA sensitivities, enhanced disease resistance, and drought tolerances may be attributed, in part, to stomata closure and induction of stress-related genes during drought induction (Qiu and Yu, 2009).

Regarding the WRKY response to pathogens, some results indicate that these genes may have a negative effect on resistance response. Isolated loss-of-function T-DNA insertion mutants and overexpressing lines for *AtWRKY48* in *Arabidopsis* have resulted in enhanced resistance of the loss-of-function mutants that was associated with an increase in salicylic acid-regulated *AtPR1* induction by the bacterial pathogen. The growth of a virulent strain of the bacterial pathogen *Pseudomonas syringae* was decreased in *wrky48* T-DNA insertion mutants. On the other hand, transgenic *WRKY48*-overexpressing plants supported enhanced growth of *P. syringae,* and the enhanced susceptibility was associated with reduced expression of defense-related *PR* genes (Xing *et al.*, 2008).

A Populus WRKY was also reported to enhance the basal resistance to fungal pathogen. Transgenic *P. tomentosa* overexpressing *P. trichocarpa* WRKY89 showed resistance to *Dothiorella gregaria,* suggesting that *PtrWRKY89* acts by accelerating the expression of PR genes in an SA-dependent defense signaling pathway (Jiang *et al.*, 2014).

Transgenic *Arabidopsis* plants overexpressing *PtrWRKY73* (*Populus trichocarpa*) showed enhanced sensitivity to *Botrytis* but enhanced resistance to *Pseudomonas syringae pv* tomato strain DC3000 through the regulation of SA-related gene expression (Duan *et al.*, 2015). A negative effect was also observed in transgenic *Nicotiana benthamiana* overexpressing *GhWRKY27a*, while exhibiting reduced resistance to *Rhizoctonia solani* infection (Yan *et al.*, 2015). According to Pandey and Somssich (2009), active targeting of WRKY genes or downstream products by pathogens and the coordinated modulation of these factors, which enable the amplification and timing of the plant’s response during infection, can explain the negative effect caused by WRKYs.

## Concluding remarks

Most studies regarding the response of transgenic plants overexpressing *WRKY* genes indicate tolerance to abiotic stresses, resistance to biotic stresses, or both. On the other hand, in studies where *WRKY* genes have been silenced, a sensitivity reaction to abiotic stresses and susceptibility to biotic stresses was observed. Some rare cases of negative regulation concerning stress were also reported.

**Figure 2:**
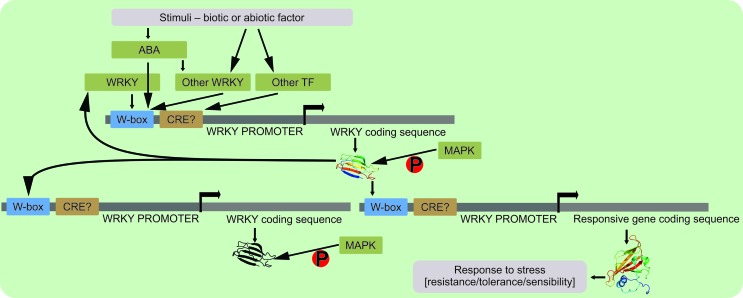
Regulatory mechanism of WRKY transcription factors. The signaling pathway starts with an environmental stimulus through ABA signaling or by *trans*-regulation, which comprise direct transcription factor activation. The *trans* regulation can occur with a WRKY member, in case of presence of a W-box, or with other TFs from different families. An auto-regulation mechanism can occur, or regulation by promoter binding of a different WRKY member. In some cases, the phosphorylation by an MAPK cascade is a determinant process for the correct WRKY protein function. When activated, WRKY TFs can regulate a different WRKY by binding its W-box sequence, or regulate some other responsive gene conferring tolerance, resistance, or sensibility toward the environmental stimuli.

The accurate regulation and fine adjustment of WRKY proteins during plant responses to stress contribute to the establishment of complex signaling networks, and the important roles of WRKY proteins in these networks make them potential candidates for conferring tolerance (Chen *et al.*, 2012) to biotic and abiotic factors. WRKYs can be auto-regulated or regulated by other WRKY members leading to an amplification of their responses (reviewed in Llorca *et al.*, 2014), or prevent WRKY activity (Eulgem 2005) in response to diverse stimuli. However, WRKY transcriptional activation can be a very complex system, since many other TFs from different families can bind to their promoters (Dong *et al.*, 2003; Shen *et al.*, 2015) (Figure 2).

In addition, considering the large number of genes of the WRKY TF family present in the genome of plant species, WRKY functions and interactions among the family members and WRKY protein kinases in the cascade of signaling need to be clarified.

The development of advanced techniques of molecular biology, such as the combined use of microarrays, immunoprecipitation assays and sequencing techniques, may on a large scale, determine the DNA WRKY protein binding sites and their interaction with other proteins in signaling pathways. In addition, the function of WRKY in plants in response to environmental challenges might be clarified.

## Supplementary material

The following online material is available for this article:

Table S1 - Studies concerning genetic transformation performed with *WRKY* genes in different plant species in response to abiotic stresses.

Table S2 - Gene transformation studies performed with *WRKY* genes in different plant species in response to biotic stresses.

Table S3 - Gene transformation studies performed with *WRKY* genes in different plant species in response to biotic and abiotic stresses.

Table S4 - Transgenic generated lines performed with WRKY genes in different plant species in response to biotic and abiotic stresses.
