# Supplementary material for: Can WRKY transcription factors help plants to overcome environmental challenges?
Source: Genet Mol Biol. 2018 Jul-Sep;41(3):533–44. doi: 10.1590/1678-4685-GMB-2017-0232 (PMC6136380; doi:10.1590/1678-4685-GMB-2017-0232)
Supplement: Supplementary file 1 [file 1415-4757-GMB-41-03-2017-0232-20180717-suppl1.pdf]

Supplementary Material to "Can WRKY transcription factors help plants to overcome environmental challenges?"

Table S1 - Studies concerning genetic transformation performed with *WRKY* genes in different plant species in response to abiotic stresses. Event (Si: Silencing, OE: Overexpression); reaction: respective phenotype observed under certain stress (T: Tolerance, S: Sensitivity)

Species	Event	Gene	Stress	Reaction	Reference
Arabidopsis thaliana	OE	*GhWRKY34*	Salt	T	Zhou *et al*., 2015
Arabidopsis thaliana	OE	*AtWRKY28/AtBHLH17*	NaCl, mannitol and oxidative stress	T	Babitha *et al*., 2012
Arabidopsis thaliana	OE	*AtWRKY18*	Salt/Osmotic	S	Chen *et al*., 2010
Arabidopsis thaliana	OE	*AtWRKY60*	Salt/Osmotic	S	Chen *et al*., 2010
Arabidopsis thaliana	OE	*AtWRKY46*	Salt	T	Ding *et al*., 2015
Arabidopsis thaliana	OE	*RtWRKY1*	Salt	T	Du *et al*., 2017
Arabidopsis thaliana	OE	*TaWRKY1*	Heat/Drought	T	He *et al*., 2016
Arabidopsis thaliana	OE	*TaWRKY33*	Heat/Drought	T	He *et al*., 2016
Arabidopsis thaliana	Si	*AtWRKY25*	Heat	S	Li *et al*., 2011
Arabidopsis thaliana	Si	*AtWRKY26*	Heat	S	Li *et al*., 2011
Arabidopsis thaliana	Si	*AtWRKY33*	Heat	S	Li *et al*., 2011
Arabidopsis thaliana	OE	*AtWRKY39*	Heat	T	Li S *et al*., 2010
Arabidopsis thaliana	OE	*VvWRKY11*	Drought	T	Liu *et al*., 2011
Arabidopsis thaliana	OE	*CmWRKY17*	Salt	S	Li *et al*., 2015
Arabidopsis thaliana	OE	*TaWRKY2*	Salt and Drought	T	Niu *et al*., 2012
Arabidopsis thaliana	OE	*TaWRKY19*	Cold, salt and drought	T	Niu *et al*., 2012
Arabidopsis thaliana	OE	*HaWRKY76*	Flooding/drought	T	Raineri *et al*., 2015
Arabidopsis thaliana	OE	*AtWRKY39*	Heat	T	Li S *et al*., 2010
Arabidopsis thaliana	OE	*CsWRKY46*	Cold	T	Zhang *et al*., 2016
Arabidopsis thaliana	OE	*GmWRKY54*	Salt	T	Zhou *et al*., 2008
Arabidopsis thaliana	OE	*GmWRKY54*	Drought	T	Zhou *et al*., 2008
Arabidopsis thaliana	OE	*GmWRKY21*	Cold	T	Zhou *et al*., 2008
Arabidopsis thaliana	OE	*GmWRKY21*	Cold	T	Zhou *et al*., 2008
Arabidopsis thaliana	OE	*GmWRKY54*	Salt/Drought	T	Zhou *et al*., 2008
Arabidopsis thaliana	OE	*GhWRKY34*	Salt	T	Zhou *et al*., 2015
Arabidopsis thaliana	OE	*GmWRKY13*	Salt/Drought	S	Zhou *et al*., 2008
Gossypium hirsutum	Si	*GhWRKY27*	Drought	T	Yan *et al*., 2015
Gossypium hirsutum	Si	*GhWRKY27a*	drought	T	Yan *et al*., 2015
Nicotiana benthamiana	OE	*GhWRKY41*	Drought/Salt	T	Chu *et al*., 2015
Nicotiana sp.	OE	*GmWRKY57B*	Drought	T	Zhang *et al*., 2008
Nicotiana benthamiana	OE	*GhWRKY17*	Drought/Salt	S	Yan *et al*., 2014
Nicotiana benthamiana	OE	*GhWRKY25*	Drought/Salt	S/T	Liu *et al*., 2015

Species	Event	Gene	Stress	Reaction	Reference
Nicotiana benthamiana	OE	*NbWRKY79*	Salt	T	Nam *et al*., 2017
Nicotiana benthamiana and *Nicotiana tabacum*	OE	*BhWRKY1*	Drought	T	Wang *et al*., 2009
Nicotiana tabacum	OE	*SlWRKY*	Salt/Drought	T	Li *et al*., 2012
Nicotiana tabacum	OE	*BcWRKY46*	Cold, salinity and Drought	T	Niu *et al*., 2012
Nicotiana tabacum	OE	*TaWRKY1*	Drought	T	Ding *et al*., 2016
Oryza sativa	OE	*OsWRKY11*	Heat/Drought	T	Wu *et al*., 2009
Oryza sativa	OE	OsWRKY74	Phosphate starvation	T	Dai *et al*., 2016
Oryza sativa	OE	*OsWRKY30*	Drought	T	Shen *et al*., 2012
Oryza sativa	OE	*OsWRKY76*	Cold	T	Yokotani *et al*., 2013
Zea mays	OE	*ZmWRKY4*	Cadmium stress	T	Hong *et al*., 2017

At – *Arabidopsis thaliana*, Bh - *Boea hygrometrica*, Bc - *Brassica campestris ssp. chinensis, Cm - Chrysanthemum, Cs - Cucurbita ssp,* Gh - *Gossypium hirsutum*, Gm - *Glycine max, Ha - Helianthus annuus, Nb - Nicotiana benthamiana,* Os - *Oryza sativa, Rt - Reaumuria trigyna,* Sl - *Solanum lycopersicum,* Ta - *Triticum aestivum*, Vv - *Vitis vinifera*, Zm - *Zea mays.*

References

Babitha KC, Ramu SV, Pruthvi V, Mahesh P, Nataraja KN and Udayakumar M (2012) Co-expression of *AtbHLH17* and *AtWRKY28* confers resistance to abiotic stress in *Arabidopsis*. Transgenic Res 22:327-341.

Chen H, Lai Z, Shi J, Xiao Y, Chen Z and Xu X (2010) Roles of *Arabidopsis* WRKY18, WRKY40 and WRKY60 transcription factors in plant responses to abscisic acid and abiotic stress. BMC Plant Biol 10:281.

Chu X, Wang C, Chen X, Lu W, Li H, Wang X, Hao L and Guo X (2015) The Cotton WRKY Gene *GhWRKY41* positively regulates salt and drought stress tolerance in transgenic *Nicotiana benthamiana*. Plos One 10:e0143022.

Dai X, Wang Y and Zhang WH (2016) *OsWRKY74*, a WRKY transcription factor, modulates tolerance to phosphate starvation in rice. J Exp Bot 67:947-960.

Ding W, Fang W, Shi S, Zhao Y, Li X and Xiao K (2016) Wheat WRKY type transcription factor gene *tawrky1* is essential in mediating drought tolerance associated with an ABA-dependent pathway. Plant Mol Biol Report 34:111-1126.

Ding ZJ, Yan JY, Li CX, Li GX, Wu YR and Zheng SJ (2015) Transcription factor WRKY46 modulates the development of *Arabidopsis* lateral roots in osmotic/salt stress conditions via regulation of ABA signaling and auxin homeostasis. Plant J 84:56-69.

Du C, Zhao P, Zhang H, Li N, Zheng L and Wang Y (2017) The *Reaumuria trigyna* transcription factor *RtWRKY1* confers tolerance to salt stress in transgenic *Arabidopsis*. J Plant Physiol 4:48-58.

He GH, Xu JY, Wang YX, Liu JM, Li PS, Chen M, Ma YZ and Xu ZS (2016) Drought-responsive WRKY transcription factor genes TaWRKY1 and *TaWRKY33* from wheat confer drought and/or heat resistance in *Arabidopsis*. BMC Plant Biol 16:116.

Hong C, Cheng D, Zhang G, Zhu D, Chen Y and Tan M (2017) The role of *ZmWRKY4* in regulating maize antioxidant defense under cadmium stress. Biochem Biophys Res Commun 482:1504-1510.

Li JB, Luan YS and Jin H (2012) The tomato *SlWRKY* gene plays an important role in the regulation of defense responses in tobacco. Biochem Biophys Res Commun 427:671-676.

Li P, Song A, Gao C, Wang L, Wang Y, Sun J, Jiang J, Chen F and Chen S (2015) Chrysanthemum WRKY gene *CmWRKY17* negatively regulates salt stress tolerance in transgenic chrysanthemum and *Arabidopsis* plants. Plant Cell Rep 34:1365-1378.

Li S, Fu Q, Chen L, Huang W and Yu D (2011) *Arabidopsis thaliana* WRKY25, WRKY26, and WRKY33 coordinate induction of plant thermo tolerance. Planta 233:1237-1252.

Li S, Zhou X, Chen L, Huang W and Yu D (2010) Functional characterization of *Arabidopsis thaliana* WRKY39 in heat stress. Mol. Cells 29, 475-483.

Liu H, Yang W, Liu D, Han Y, Zhang A and Li S (2011) Ectopic expression of a grapevine transcription factor *VvWRKY11* contributes to osmotic stress tolerance in *Arabidopsis.* Mol Biol Rep 38:417-427.

Liu X, Song Y, Xing F, Wang N, Wen F and Zhu C (2015) *GhWRKY25*, a group I WRKY gene from cotton, confers differential tolerance to abiotic and biotic stresses in transgenic *Nicotiana benthamiana*. Protoplasma 253:1265-1281.

Nam TN, Thia LH, Mai DS and Tuan NV (2017) Overexpression of *NbWRKY79* enhances salt stress tolerance in *Nicotiana benthamiana*. Acta Physiol Plant 39:121.

Niu CF, Wei W, Zhou QY, Tian AG, Hao YJ, Zhang WK, Ma B, Lin Q, Zhang ZB, Zhang JS, *et al*., (2012) Wheat WRKY genes *TaWRKY2* and *TaWRKY19* regulate abiotic stress tolerance in transgenic *Arabidopsis* plants. Plant Cell Environ 35:1156-1170.

Raineri J, Ribichich KF and Chan RL (2015) The sunflower transcription factor *HaWRKY76* confers drought and flood tolerance to *Arabidopsis thaliana* plants without yield penalty. Plant Cell Rep 34:2065-2080.

Shen H, Liu C, Zhang Y and Meng X (2012) OsWRKY30 is activated by MAP kinases to confer drought tolerance in rice. Plant Mol Biol 80:241-253.

Wang Z, Zhu Y, Wang L, Liu X, Liu Y, Phillips J and Deng X (2009) A WRKY transcription factor participates in dehydration tolerance in *Boea hygrometrica* by binding to the W-box elements of the galactinol synthase (BhGolS1) promoter. Planta 230:1155-1166.

Wu X, Shiroto Y, Kishitani S, Ito Y and Toriyama K (2009) Enhanced heat and drought tolerance in transgenic rice seedlings overexpressing *OsWRKY11* under the control of HSP101 promoter. Plant Cell Rep 28:21-30.

Yan H, Jia H, Chen X, Hao L, An H and Guo X (2014) The cotton WRKY transcription factor *GhWRKY17* functions in drought and salt stress in transgenic *Nicotiana benthamiana* through ABA signaling and the modulation of reactive oxygen species production. Plant Cell Physiol 55:2060-2076.

Yan Y, Jia H, Wang F, Wang C, Shuchang Liu and Xingqi Guo (2015) Overexpression of *GhWRKY27a* reduces tolerance to drought stress and resistance to *Rhizoctonia solani* infection in transgenic *Nicotiana benthamiana*. Front Physiol 6:265.

Yokotani N, Sato Y, Tanabe S, Chujo T, Shimizu T, Okada K, Yamane H, Shimono M, Sugano S, Takatsuji H, *et al*. (2013) WRKY76 is a rice transcriptional repressor playing opposite roles in blast disease resistance and cold stress tolerance. J Exp Bot 64:5085-5097.

Zhang L, Wang X, Bi Y, Zhang C, Fan YL and Wang L (2008) Isolation and functional analysis of transcription factor *GmWRKY57B* from soybean. Chin Sci Bull 53:3538-3545.

Zhang Y, Yu H, Yang X, Li Q, Ling J, Wang H, Gu X, Huang S and Jiang W (2016) *CsWRKY46*, a WRKY transcription factor from cucumber, confers cold resistance in transgenic-plant by regulating a set of cold-stress responsive genes in an ABA-dependent manner. Plant Physiol Biochem 108:478-487.

Zhou L, Wang NN, Gong SY, Lu Rui, Li Y and Li XB (2015) Overexpression of a cotton (*Gossypium hirsutum*) WRKY gene, *GhWRKY34*, in *Arabidopsis* enhances salt-tolerance of the transgenic plants. Plant Physiol Biochem 96:311-320.

Zhou QY, Tian AG, Zou HF, Xie ZM, Lei G, Huang J, Wang CM, Wang HW, Zhang JS and Chen SY (2008) Soybean WRKY-type transcription factor genes, *GmWRKY13*, *GmWRKY21*, and *GmWRKY54*, confer differential tolerance to abiotic stresses in transgenic *Arabidopsis* plants. Plant Biotechnol J 6:486-503.
