# Supplementary material for: Can WRKY transcription factors help plants to overcome environmental challenges?
Source: Genet Mol Biol. 2018 Jul-Sep;41(3):533–44. doi: 10.1590/1678-4685-GMB-2017-0232 (PMC6136380; doi:10.1590/1678-4685-GMB-2017-0232)
Supplement: Supplementary file 2 [file 1415-4757-GMB-41-03-2017-0232-20180717-suppl2.pdf]

Supplementary Material to "Can WRKY transcription factors help plants to overcome environmental challenges?"

Table S2 - Gene transformation studies performed with *WRKY* genes in different plant species in response to biotic stresses. Event (Si: Silencing, OE: Overexpression); reaction: respective phenotype observed under certain stress (R: Resistance, S: Susceptibility)

Species	Event	Gene	Stress	Reaction	Reference
Arabidopsis thaliana	OE	*PtrWRKY89*	*Botrytis cinerea*	S	Jiang *et al*., 2016
Arabidopsis thaliana	OE	*PtrWRKY73*	*Botrytis cinerea/Pseudomonas syringae pv*	S/R	Duan *et al*., 2015
Arabidopsis thaliana	Si	*AtWRKY75*	*Pseudomonas syringae*	S	Encinas-Villarejo *et al*., 2009
Arabidopsis thaliana	OE	*FaWRKY1*	*Pseudomonas syringae*	R	Encinas-Villarejo *et al*., 2009
Arabidopsis thaliana	OE	*AtWRKY75*	*Pseudomonas syringae*	R	Encinas-Villarejo *et al*., 2009
Arabidopsis thaliana	OE	*OsWRKY23*	*Pseudomanas syringae*	R	Shaojuan *et al*., 2009
Arabidopsis thaliana	Si	*AtWRKY48*	*Pseudomonas syringae*	R	Xing *et al*., 2008
Arabidopsis thaliana	OE	*OsWRKY6*	*Xanthomonas oryzae pv. oryzae*	R	Hwang *et al*., 2011
Arabidopsis thaliana	OE	*VqWRKY52*	*Pseudomonas syringae*	R	Wang *et al*., 2017
Arabidopsis thaliana	OE	*VqWRKY52*	*Botrytis cinerea*	S	Wang *et al*., 2017
Arabidopsis thaliana	Si	*AtWRKY57*	*Botrytis cinerea*	R	Jiang and Yu 2016
Arabidopsis thaliana	Si	*AtWRKY23*	*Heterodera schachtii*	R	Grunewald *et al*., 2008
Arabidopsis thaliana	OE	*OsWRKY23*	*Pyricularia oryzae Cav*	R	Shaojuan *et al*., 2009
Arabidopsis thaliana	OE	*OsWRKY23*	*Pseudomonas syringae*	R	Shaojuan *et al*., 2009
Capsicum annuum	Si	*CaWRKY58*	*Ralstonia solanacearum*	S	Wang *et al*., 2012
Gossypium hirsutum	OE	*GhWRKY15*	*Colletotrichum gossypi/Phytophthora parasitica*	R/R	Yu *et al*., 2012
Nicotiana attenuata	Si	*NaWRKY3*	*Manduca sexta*	S	Skibbe *et al*., 2008
Nicotiana attenuata	Si	*NaWRKY6*	*Manduca sexta*	S	Skibbe *et al*., 2008
Nicotiana benthamiana	OE	*GhWRKY44*	*Ralstonia solanacearum/Rhizoctonia solani*	R/R	Li *et al*., 2014
Nicotiana sp.	OE	*MdWRKY1*	*Phytophthoraparasiticavar.nicotianae*	R	Fan *et al*., 2011
Oryza sativa	OE	*OsWRKY45-1*	*Magnaporthe grisea*	R	Tao *et al*., 2009
Oryza sativa	OE	*OsWRKY45-2*	*Magnaporthe grisea*	R	Tao *et al*., 2009
Oryza sativa	OE	*OsWRKY62*	*Xanthomonas oryzae pv. oryzae*	R	Peng *et al*., 2010
Oryza sativa	OE	OsWRKY28	*Xanthomonas oryzae pv. oryzae*	R	Peng *et al*., 2010
Oryza sativa	OE	OsWRKY71	*Xanthomonas oryzae pv. oryzae*	R	Peng *et al*., 2010
Oryza sativa	OE	OsWRKY76	*Xanthomonas oryzae pv. oryzae*	R	Peng *et al*., 2010
Oryza sativa	Si	OsWRKY22	*Magnaporthe oryzae*	S	Abbruscato *et al*., 2012
Oryza sativa	OE	*OsWRKY22*	*Magnaporthe oryzae*	R	Abbruscato *et al*., 2012
Oryza sativa	OE	*OsWRKY80*	*Rhizoctonia solani*	R	Peng *et al*., 2016
Populus tomentosa	OE	*PtrWRKY89*	*Dothiorella gregaria*	R	Jiang *et al*., 2014
Solanum lycopersicum	Si	*SlWRKY70*	*Meloidogyne javanica*	S	Atamian *et al*., 2012
Solanum lycopersicum	Si	*SlWRKY70*	*Macrosiphum euphorbiae*	S	Atamian *et al*., 2012

At - *Arabidopsis thaliana*, Ca - *Capsicum annuum*, Fa - *Fragaria* x *ananassa*, Gh - *Gossypium hirsutum*, Md - *Malus domestica*, Na - *Nicotiana attenuata*, Os - *Oryza sativa*, *Ptr - Populus trichocarpa, Sl - Solanum lycopersicum, Vq - Vitis quinquangularis.*

References

Abbruscato P, Nepusz T, Mizzi L, Del Corvo M, Morandini P, Fumasoni I, Michel C, Paccanaro A, Guiderdoni E, Schaffrath U, *et al*. (2012) *OsWRKY22*, a monocot WRKY gene, plays a role in the resistance response to blast. Mol Plant Pathol 13:828–841.

Atamian HS, Eulgem T and Kaloshian I (2012) SlWRKY70 is required for Mi-1-mediated resistance to aphids and nematodes in tomato. Planta 235:299-309.

Duan Y, Jiang Y, Ye S, Karim A, Ling Z, He Y, Yang S and Luo K (2015) *PtrWRKY73,* a salicylic acid-inducible poplar WRKY transcription factor, is involved in disease resistance in *Arabidopsis thaliana*. Plant Cell Rep 34:831-841.

Encinas-Villarejo S, Maldonado AM, Amil-Ruiz F, de los Santos B, Romero F, Pliego-Alfaro F, Muñoz-Blanco J and Caballero JL (2009) Evidence for a positive regulatory role of strawberry (Fragaria x ananassa) *FaWRKY1* and *Arabidopsis AtWRKY75* proteins in resistance. J Exp Bot 60:3043-3065.

Fan H, Wang F, Gao H, Wang L, Xu J and Zhao Z (2011) Pathogen-induced *MdWRKY1* in "Qinguan" apple enhances disease resistance. J Plant Biol 54:150-158.

Grunewald W, Karimi M, Wieczorek K, Van de Cappelle E, Wischnitzki E, Grundler F, Inzé D, Beeckman T and Gheysen G (2008) A role for AtWRKY23 in feeding site establishment of plant-parasitic nematodes. Plant Physiol 148:358-368.

Hwang SH, Yie SW and Hwang DJ (2011) Heterologous expression of *OsWRKY6* gene in *Arabidopsis* activates the expression of defense related genes and enhances resistance to pathogens. Plant Sci 181:316–323.

Jiang Y and Yu D (2016) The WRKY57 transcription factor affects the expression of jasmonate ZIM-Domain genes transcriptionally to compromise *Botrytis cinerea* resistance. Plant Physiol 171:2771-82.

Jiang Y, Duan Y, Yin J, Ye S, Zhu J, Zhang F, Lu W, Fan D and Luo K (2014) Genome-wide identification and characterization of the Populus WRKY transcription factor family and analysis of their expression in response to biotic and abiotic stresses. J Exp Bot 65:6629-6644.

Jiang Y, Guo L, Liu R, Jiao B, Zhao X, Ling Z and Luo K (2016) Overexpression of Poplar *PtrWRKY89* in transgenic *Arabidopsis* leads to a reduction of disease resistance by regulating defense related genes in salicylate and jasmonate dependent signaling. PLoS One 11:0149137.

Li J, Wang J, Wang N, Guo X and Gao Z (2014) *GhWRKY44*, a WRKY transcription factor of cotton, mediates defense responses to pathogen infection in transgenic *Nicotiana benthamiana*. Plant Cell Tissue Organ Cult 121:127-140.

Peng X, Wang H, Jang JC, Xiao T, He H, Jiang D and Tang X (2016) *OsWRKY80-OsWRKY4* module as a positive regulatory circuit in rice resistance against *Rhizoctonia solani*. Rice 9:1-14.

Peng Y, Bartley L, Canlas P and Ronald P (2010) OsWRKYIIa transcription factors modulate rice innate immunity. Rice 3:36-42.

Shaojuan SJ, Zhou X, Song Y and Yu D (2009) Heterologous expression of *OsWRKY23* gene enhances pathogen defense and dark-induced leaf senescence in *Arabidopsis*. Plant Growth Regul 58:181-190.

Skibbe M, Qu N, Galis I and Baldwin IT (2008) Induced plant defenses in the natural environment: *Nicotiana attenuata WRKY3* and *WRKY6* Coordinate responses to herbivory. Plant Cell 20:1984-2000.

Tao Z, Liu H, Qiu D, Zhou Y, Li X, Xu C and Wang S (2009) A pair of allelic WRKY genes play opposite roles in rice-bacteria interactions. Plant Physiol 151:936-948.

Wang X, Guo R, Tu M, Wang D, Guo C, Wan R, Li Z and Wang X (2017) Ectopic expression of the wild grape WRKY transcription factor *VqWRKY52* in *Arabidopsis thaliana* enhances resistance to the biotrophic pathogen powdery mildew but not to the necrotrophic pathogen *Botrytis cinerea*. Front Plant Sci 31:97.

Wang Y, Dang F, Liu Z, Wang X, Eulgem T, Lai Y, Yu L, She J, Shi Y, Lin J, *et al*. (2012) *CaWRKY58*, encoding a group I WRKY transcription factor of *Capsicum annuum,* negatively regulates resistance to *Ralstonia solanacearum* infection. Mol Plant Pathol 14:131-144.

Xing DH, Lai ZB, Zheng ZY, Vinod KM, Fan BF and Chen ZX (2008) Stress-and pathogen-induced *Arabidopsis* WRKY48 is a transcriptional activator that represses plant basal defense. Mol Plant 1:459-470.

Yu F, Huaxia Y, Lu W, Wu C, Cao X and Guo X (2012) *GhWRKY15*, a member of the WRKY transcription factor family identified from cotton (*Gossypium hirsutum* L.), is involved in disease resistance and plant development. BMC Plant Biol 12:144.
