# Supplementary material for: Can WRKY transcription factors help plants to overcome environmental challenges?
Source: Genet Mol Biol. 2018 Jul-Sep;41(3):533–44. doi: 10.1590/1678-4685-GMB-2017-0232 (PMC6136380; doi:10.1590/1678-4685-GMB-2017-0232)
Supplement: Supplementary file 3 [file 1415-4757-GMB-41-03-2017-0232-20180717-suppl3.pdf]

Supplementary Material to "Can WRKY transcription factors help plants to overcome environmental challenges?"

Table S3 - Gene transformation studies performed with *WRKY* genes in different plant species in response to biotic and abiotic stresses. Event (Si: silence, OE: overexpression); reaction: their phenotype observed against certain stress (T: Tolerance, R: Resistance, S: Sensitivity or Susceptibility) and the reference source

Species	Event	Gene	Stress	Reaction	Reference
Arabidopsis thaliana	OE	*VpWRKY1*	*Erysiphe cichoracearum*/Salt	R/T	Li *et al*., 2010
Arabidopsis thaliana	OE	*VpWRKY2*	*Erysiphe cichoracearum*/Salt and Cold	R/T	Li *et al*., 2010
Arabidopsis thaliana	OE	*OsWRKY45*	*Pseudomonas syringae tomato* / Salt and drought	R /T	Qiu and Yu, 2009
Nicotiana benthamiana	OE	*GhWRKY27*	*Rhizoctonia solani*/Drougth	S/S	Yan *et al*., 2015
Nicotiana tabacum	OE	*VpWRKY3*	*Ralstonia solanacearum* /Salt	R/T	Zhu *et al*., 2012
Oryza sativa	OE	*OsWRKY89*	*Magnaporthe griseae* and *Sogatella furcifera*/UV-B	R/T	Wang *et al*., 2017
Oryza sativa	Si	*OsWRKY89*	*Magnaporthe griseae* and *Sogatella furcifera*/UV-B	S/S	Wang *et al*., 2017
Oryza sativa	OE	*OsWRKY76*	*Magnaporthe oryzae*/Cold	T/S	Yokotani *et al*., 2013

Gh – *Gossypium hisutum*, Os - *Oryza sativa*, Vp – *Vitis pseudoreticulata*.

References

Li H, Xu Y, Xiao Y, Zhu Z, Xie X, Zhao H and Wang Y (2010) Expression and functional analysis of two genes encoding transcription factors, *VpWRKY1* and *VpWRKY2*, isolated from Chinese wild *Vitis pseudoreticulata*. Planta 232:1325-1337.

Qiu Y and Yu D (2009) Over-expression of the stress-induced *OsWRKY45* enhances disease resistance and drought tolerance in *Arabidopsis*. Environ Exp Bot 65:35-47.

Wang X, Guo R, Tu M, Wang D, Guo C, Wan R, Li Z and Wang X (2017) Ectopic expression of the wild grape WRKY transcription factor *VqWRKY52* in *Arabidopsis thaliana* enhances resistance to the biotrophic pathogen powdery mildew but not to the necrotrophic pathogen *Botrytis cinerea*. Front Plant Sci 31:97.

Yan Y, Jia H, Wang F, Wang C, Shuchang Liu and Xingqi Guo (2015) Overexpression of *GhWRKY27a* reduces tolerance to drought stress and resistance to *Rhizoctonia solani* infection in transgenic *Nicotiana benthamiana*. Front Physiol 6:265.

Yokotani N, Sato Y, Tanabe S, Chujo T, Shimizu T, Okada K, Yamane H, Shimono M, Sugano S, Takatsuji H, *et al*. (2013) WRKY76 is a rice transcriptional repressor playing opposite roles in blast disease resistance and cold stress tolerance. J Exp Bot 64:5085-5097.

Zhu Z, Shi J, Cao J, He M and Wang Y (2012) *VpWRKY3*, a biotic and abiotic stress-related transcription factor from the Chinese wild *Vitis pseudoreticulata*. Plant Cell Rep 31:2109-2120.
