# Supplementary material for: Can WRKY transcription factors help plants to overcome environmental challenges?
Source: Genet Mol Biol. 2018 Jul-Sep;41(3):533–44. doi: 10.1590/1678-4685-GMB-2017-0232 (PMC6136380; doi:10.1590/1678-4685-GMB-2017-0232)
Supplement: Supplementary file 4 [file 1415-4757-GMB-41-03-2017-0232-20180717-suppl4.pdf]

Supplementary Material to "Can WRKY transcription factors help plants to overcome environmental challenges?"

Table S4 – Transgenic generated lines performed with *WRKY* genes in different plant species in response to biotic and abiotic stresses. Event (Si: silence, OE: overexpression) reaction - their phenotype observed against certain stress (T: Tolerance, R: Resistance, S: Sensitivity or Susceptibility) and the reference source

Species	Event	Gene	Stress	Reaction	Reference
Arabidopsis thaliana	OE	*GhWRKY34*	Salt	T	Zhou *et al*., 2015
Arabidopsis thaliana	OE	*RtWRKY1*	Salt	T	Du *et al*., 2017
Arabidopsis thaliana	OE	*TaWRKY1*	Heat/Drought	T	He *et al*., 2016
Arabidopsis thaliana	OE	*TaWRKY33*	Heat/Drought	T	He *et al*., 2016
Arabidopsis thaliana	OE	*VvWRKY11*	Drought	T	Liu *et al*., 2011
Arabidopsis thaliana	OE	*CmWRKY17*	Salt	S	Li *et al*., 2015
Arabidopsis thaliana	OE	*TaWRKY2*	Salt and Drought	T	Niu *et al*., 2012
Arabidopsis thaliana	OE	*TaWRKY19*	Cold, salt and drought	T	Niu *et al*., 2012
Arabidopsis thaliana	OE	*HaWRKY76*	Flooding/drought	T	Raineri *et al*., 2015
Arabidopsis thaliana	OE	*CsWRKY46*	Cold	T	Zhang *et al*., 2016
Arabidopsis thaliana	OE	*GmWRKY54*	Salt	T	Zhou *et al*., 2008
Arabidopsis thaliana	OE	*GmWRKY54*	Drought	T	Zhou *et al*., 2008
Arabidopsis thaliana	OE	*GmWRKY21*	Cold	T	Zhou *et al*., 2008
Arabidopsis thaliana	OE	*GmWRKY21*	Cold	T	Zhou *et al*., 2008
Arabidopsis thaliana	OE	*GmWRKY54*	Salt/Drought	T	Zhou *et al*., 2008
Arabidopsis thaliana	OE	*GhWRKY34*	Salt	T	Zhou *et al*., 2015
Arabidopsis thaliana	OE	*GmWRKY13*	Salt/Drought	S	Zhou *et al*., 2008
Arabidopsis thaliana	OE	*PtrWRKY89*	*Botrytis cinerea*	S	Jiang *et al*., 2016
Arabidopsis thaliana	OE	*PtrWRKY73*	*Botrytis cinerea/Pseudomonas syringae pv*	S/R	Duan *et al*., 2015
Arabidopsis thaliana	OE	*FaWRKY1*	*Pseudomonas syringae*	R	Encinas-Villarejo *et al*., 2009
Arabidopsis thaliana	OE	*OsWRKY23*	*Pseudomanas syringae*	R	Shaojuan *et al*., 2009
Arabidopsis thaliana	OE	*OsWRKY6*	*Xanthomonas oryzae pv. oryzae*	R	Hwang *et al*., 2011
Arabidopsis thaliana	OE	*VqWRKY52*	*Pseudomonas syringae*	R	Wang *et al*., 2017
Arabidopsis thaliana	OE	*VqWRKY52*	*Botrytis cinerea*	S	Wang *et al*., 2017
Arabidopsis thaliana	OE	*OsWRKY23*	*Pyricularia oryzae Cav*	R	Shaojuan *et al*., 2009

Species	Event	Gene	Stress	Reaction	Reference
Arabidopsis thaliana	OE	*OsWRKY 23*	*Pseudomonas syringae*	R	Shaojuan *et al.*, 2009
Arabidopsis thaliana	OE	*VpWRKY 1*	*Erysiphe cichoracearum*/Salt	R/T	Li H *et al.*, 2010
Arabidopsis thaliana	OE	*VpWRKY 2*	*Erysiphe cichoracearum*/Salt and Cold	R/T	Li H *et al.*, 2010
Arabidopsis thaliana	OE	*OsWRKY 45*	*Pseudomonas syringae tomato* / Salt and drought	R /T	Qiu and Yu, 2009
Capsicum annuum	Si	*CaWRK Y58*	*Ralstonia solanacearum*	S	Wang *et al.*, 2012
Gossypium hirsutum	Si	*GhWRK Y27a*	drought	T	Yan *et al.*, 2015
Gossypium hirsutum	Si	*GhWRK Y27*	Drought	T	Yan *et al.*, 2015
Nicotiana benthamiana	OE	*GhWRK Y17*	Drought/Salt	S	Yan *et al.*, 2014
Nicotiana benthamiana	OE	*GhWRK Y41*	Drought/Salt	T	Chu *et al.*, 2015
Nicotiana benthamiana	OE	*GhWRK Y25*	Drought/Salt	S/T	Liu *et al.*, 2015
Nicotiana benthamiana	OE	*GhWRK Y44*	*Ralstonia solanacearum/Rhizoctonia solani*	R/R	Li *et al.*, 2014
Nicotiana benthamiana	OE	*GhWRK Y27*	*Rhizoctonia solani/*Drougth	S/S	Yan *et al.*, 2015
Nicotiana benthamiana and *Nicotiana tabacum*	OE	*BhWRKY 1*	Drought	T	Wang *et al.*, 2009
Nicotiana sp.	OE	*GmWRK Y57B*	Drought	T	Zhang *et al.*, 2008
Nicotiana sp.	OE	*MdWRK Y1*	*Phytophthora parasitica var. nicotianae*	R	Fan *et al.*, 2011
Nicotiana tabacum	OE	*SlWRKY*	Salt/Drought	T	Li *et al.*, 2012
Nicotiana tabacum	OE	*BcWRKY 46*	Cold, salinity and Drought	T	Niu *et al.*, 2012
Nicotiana tabacum	OE	*TaWRKY 1*	Drought	T	Ding *et al.*, 2016
Nicotiana tabacum	OE	*VpWRKY 3*	*Ralstonia solanacearum /*Salt	R/T	Zhu *et al.*, 2012
Populus tomentosa	OE	*PtrWRK Y89*	*Dothiorella gregaria*	R	Jiang *et al.*, 2014

At - *Arabidopsis thaliana, Bc - Brassica campestris ssp. chinensis, Bh - Boea hygrometrica,* Ca - *Capsicum annuum, Cm - Chrysanthemum, Cs - Cucurbita ssp,* Fa - *Fragaria* x *ananassa,* Gh - *Gossypium hirsutum, Gm - Glycine max, Ha - Helianthus annuus,* Md - *Malus domestica, Nb - Nicotiana benthamiana,* Os - *Oryza sativa, Ptr - Populus trichocarpa, Rt - Reaumuria trigyna, Sl - Solanum lycopersicum, Ta - Triticum aestivum,* Vp - *Vitis pseudoreticulata, Vq - Vitis quinquangularis, Vv - Vitis vinífera.*

References

Chu X, Wang C, Chen X, Lu W, Li H, Wang X, Hao L and Guo X (2015) The Cotton WRKY Gene *GhWRKY41* positively regulates salt and drought stress tolerance in transgenic *Nicotiana benthamiana*. Plos One 10:e0143022.

Ding W, Fang W, Shi S, Zhao Y, Li X and Xiao K (2016) Wheat WRKY type transcription factor gene *tawrky1* is essential in mediating drought tolerance associated with an ABA-dependent pathway. Plant Mol Biol Report 34:111-1126.

Du C, Zhao P, Zhang H, Li N, Zheng L and Wang Y (2017) The *Reaumuria trigyna* transcription factor *RtWRKY1* confers tolerance to salt stress in transgenic *Arabidopsis*. J Plant Physiol 4:48-58.

Duan Y, Jiang Y, Ye S, Karim A, Ling Z, He Y, Yang S and Luo K (2015) *PtrWRKY73*, a salicylic acid-inducible poplar WRKY transcription factor, is involved in disease resistance in *Arabidopsis thaliana*. Plant Cell Rep 34:831-841.

Encinas-Villarejo S, Maldonado AM, Amil-Ruiz F, de los Santos B, Romero F, Pliego-Alfaro F, Muñoz-Blanco J and Caballero JL (2009) Evidence for a positive regulatory role of strawberry (Fragaria x ananassa) *FaWRKY1* and *Arabidopsis AtWRKY75* proteins in resistance. J Exp Bot 60:3043-3065.

Fan H, Wang F, Gao H, Wang L, Xu J and Zhao Z (2011) Pathogen-induced *MdWRKY1* in "Qinguan" apple enhances disease resistance. J Plant Biol 54:150-158.

He GH, Xu JY, Wang YX, Liu JM, Li PS, Chen M, Ma YZ and Xu ZS (2016) Drought-responsive WRKY transcription factor genes TaWRKY1 and *TaWRKY33* from wheat confer drought and/or heat resistance in *Arabidopsis*. BMC Plant Biol 16:116.

Hwang SH, Yie SW and Hwang DJ (2011) Heterologous expression of *OsWRKY6* gene in *Arabidopsis* activates the expression of defense related genes and enhances resistance to pathogens. Plant Sci 181:316–323.

Jiang Y, Duan Y, Yin J, Ye S, Zhu J, Zhang F, Lu W, Fan D and Luo K (2014) Genome-wide identification and characterization of the Populus WRKY transcription factor family and analysis of their expression in response to biotic and abiotic stresses. J Exp Bot 65:6629-6644.

Jiang Y, Guo L, Liu R, Jiao B, Zhao X, Ling Z and Luo K (2016) Overexpression of Poplar *PtrWRKY89* in transgenic *Arabidopsis* leads to a reduction of disease resistance by regulating defense related genes in salicylate and jasmonate dependent signaling. PLoS One 11:0149137.

Li H, Xu Y, Xiao Y, Zhu Z, Xie X, Zhao H and Wang Y (2010) Expression and functional analysis of two genes encoding transcription factors, *VpWRKY1* and *VpWRKY2,* isolated from Chinese wild *Vitis pseudoreticulata*. Planta 232:1325-1337.

Li J, Wang J, Wang N, Guo X and Gao Z (2014) *GhWRKY44,* a WRKY transcription factor of cotton, mediates defense responses to pathogen infection in transgenic *Nicotiana benthamiana*. Plant Cell Tissue Organ Cult 121:127-140.

Li JB, Luan YS and Jin H (2012) The tomato *SlWRKY* gene plays an important role in the regulation of defense responses in tobacco. Biochem Biophys Res Commun 427:671-676.

Li P, Song A, Gao C, Wang L, Wang Y, Sun J, Jiang J, Chen F and Chen S (2015) Chrysanthemum WRKY gene *CmWRKY17* negatively regulates salt stress tolerance in transgenic chrysanthemum and *Arabidopsis* plants. Plant Cell Rep 34:1365-1378.

Liu H, Yang W, Liu D, Han Y, Zhang A and Li S (2011) Ectopic expression of a grapevine transcription factor *VvWRKY11* contributes to osmotic stress tolerance in *Arabidopsis.* Mol Biol Rep 38:417-427.

Liu X, Song Y, Xing F, Wang N, Wen F and Zhu C (2015) *GhWRKY25*, a group I WRKY gene from cotton, confers differential tolerance to abiotic and biotic stresses in transgenic *Nicotiana benthamiana*. Protoplasma 253:1265-1281.

Niu CF, Wei W, Zhou QY, Tian AG, Hao YJ, Zhang WK, Ma B, Lin Q, Zhang ZB, Zhang JS, *et al*., (2012) Wheat WRKY genes *TaWRKY2* and *TaWRKY19* regulate abiotic stress tolerance in transgenic *Arabidopsis* plants. Plant Cell Environ 35:1156-1170.

Qiu Y and Yu D (2009) Over-expression of the stress-induced *OsWRKY45* enhances disease resistance and drought tolerance in *Arabidopsis*. Environ Exp Bot 65:35-47.

Raineri J, Ribichich KF and Chan RL (2015) The sunflower transcription factor *HaWRKY76* confers drought and flood tolerance to *Arabidopsis thaliana* plants without yield penalty. Plant Cell Rep 34:2065-2080.

Shaojuan SJ, Zhou X, Song Y and Yu D (2009) Heterologous expression of *OsWRKY23* gene enhances pathogen defense and dark-induced leaf senescence in *Arabidopsis*. Plant Growth Regul 58:181-190.

Wang X, Guo R, Tu M, Wang D, Guo C, Wan R, Li Z and Wang X (2017) Ectopic expression of the wild grape WRKY transcription factor *VqWRKY52* in *Arabidopsis thaliana* enhances resistance to the biotrophic pathogen powdery mildew but not to the necrotrophic pathogen *Botrytis cinerea*. Front Plant Sci 31:97.

Wang Y, Dang F, Liu Z, Wang X, Eulgem T, Lai Y, Yu L, She J, Shi Y, Lin J, *et al*. (2012) *CaWRKY58*, encoding a group I WRKY transcription factor of *Capsicum annuum*, negatively regulates resistance to *Ralstonia solanacearum* infection. Mol Plant Pathol 14:131-144.

Wang Z, Zhu Y, Wang L, Liu X, Liu Y, Phillips J and Deng X (2009) A WRKY transcription factor participates in dehydration tolerance in *Boea hygrometrica* by binding to the W-box elements of the galactinol synthase (BhGolS1) promoter. Planta 230:1155-1166.

Yan H, Jia H, Chen X, Hao L, An H and Guo X (2014) The cotton WRKY transcription factor *GhWRKY17* functions in drought and salt stress in transgenic *Nicotiana benthamiana* through ABA signaling and the modulation of reactive oxygen species production. Plant Cell Physiol 55:2060-2076.

Yan Y, Jia H, Wang F, Wang C, Shuchang Liu and Xingqi Guo (2015) Overexpression of *GhWRKY27a* reduces tolerance to drought stress and resistance to *Rhizoctonia solani* infection in transgenic *Nicotiana benthamiana*. Front Physiol 6:265.

Zhang L, Wang X, Bi Y, Zhang C, Fan YL and Wang L (2008) Isolation and functional analysis of transcription factor *GmWRKY57B* from soybean. Chin Sci Bull 53:3538-3545.

Zhang Y, Yu H, Yang X, Li Q, Ling J, Wang H, Gu X, Huang S and Jiang W (2016) *CsWRKY46*, a WRKY transcription factor from cucumber, confers cold resistance in transgenic-plant by regulating a set of cold-stress responsive genes in an ABA-dependent manner. Plant Physiol Biochem 108:478-487.

Zhou L, Wang NN, Gong SY, Lu Rui, Li Y and Li XB (2015) Overexpression of a cotton (*Gossypium hirsutum*) WRKY gene, *GhWRKY34*, in *Arabidopsis* enhances salt-tolerance of the transgenic plants. Plant Physiol Biochem 96:311-320.

Zhou QY, Tian AG, Zou HF, Xie ZM, Lei G, Huang J, Wang CM, Wang HW, Zhang JS and Chen SY (2008) Soybean WRKY-type transcription factor genes, *GmWRKY13*, *GmWRKY21*, and *GmWRKY54*, confer differential tolerance to abiotic stresses in transgenic *Arabidopsis* plants. Plant Biotechnol J. 6:486-503.

Zhu Z, Shi J, Cao J, He M and Wang Y (2012) *VpWRKY3*, a biotic and abiotic stress-related transcription factor from the Chinese wild *Vitis pseudoreticulata*. Plant Cell Rep 31:2109-2120.
